# Rebuilding a realistic corticostriatal “social network” from dissociated cells

**DOI:** 10.3389/fnsys.2015.00063

**Published:** 2015-04-20

**Authors:** Marianela Garcia-Munoz, Eddy Taillefer, Reuven Pnini, Catherine Vickers, Jonathan Miller, Gordon W. Arbuthnott

**Affiliations:** ^1^Brain Mechanisms for Behaviour Unit, Okinawa Institute of Science and Technology Graduate UniversityOkinawa, Japan; ^2^Physics and Biology Unit, Okinawa Institute of Science and Technology Graduate UniversityOnna-son, Japan; ^3^Neurobiology Unit, Okinawa Institute of Science and Technology Graduate UniversityOkinawa, Japan

**Keywords:** neuronal cultures, striatal neurons, cortical neurons, synaptic connections, interneurons, mutual information

## Abstract

Many of the methods available for the study of cortical influences on striatal neurons have serious problems. *In vivo* the connectivity is so complex that the study of input from an individual cortical neuron to a single striatal cell is nearly impossible. Mixed corticostriatal cultures develop many connections from striatal cells to cortical cells, in striking contrast to the fact that only connections from cortical cells to striatal cells are present *in vivo*. Furthermore, interneuron populations are over-represented in organotypic cultures. For these reasons, we have developed a method for growing cortical and striatal neurons in separated compartments that allows cortical neurons to innervate striatal cells in culture. The method works equally well for acutely dissociated or cryopreserved neurons and allows a number of manipulations that are not otherwise possible. Either cortical or striatal compartments can be transfected with channel rhodopsins. The activity of both areas can be recorded in multielectrode arrays or individual patch recordings from pairs of cells. Finally, corticostriatal connections can be severed acutely. This procedure enables determination of the importance of corticostriatal interaction in the resting pattern of activity. These cultures also facilitate development of sensitive analytical network methods to track connectivity.

## Introduction

Dopamine, the neurotransmitter lost in Parkinson's disease (Hornykiewicz, 1966), is thought to act on synaptic efficacy in striatum (Wickens et al., 1996); however, studies on the strength of corticostriatal synapses have yielded widely disparate results (Wang et al., 2006; Fieblinger et al., 2014). Reconstruction of a functioning corticostriatal system *in vitro* might enable reliable and reproducible observations. Although there have been several attempts at *in vitro* reconstruction, they all suffer from severe disadvantages.

The obvious way to look at the effects of dopamine on plasticity is *in vivo*, and this has been accomplished in a heroic series of experiments. Once animals were trained to self-stimulate through electrodes in the medial forebrain bundle, stimulation similar to that experienced by the animals was observed to increase intracellular size of postsynaptic potentials in striatal cells (Reynolds et al., 2001). Dopamine receptor antagonists blocked the effect. This positive result notwithstanding, many questions remained. Is the action pre- or post-synaptic? How does the observed facilitation interact with the synchrony imposed by the anaesthetic? A method that does not involve anaesthesia could help resolve some of these questions, as would a preparation that allowed easier access to cells to enable patch clamp recording and better control of the membrane potential.

When the earliest computer models of the corticostriatal system were conceived there were no known rules to connect cortical areas with striatal ones. It was evident that all cortical areas projected to striatum and lacked a point-to-point connectivity; therefore the simplest “all to all” connectivity pattern was assumed. As more was learned about anatomical details, a massive convergence of many cortical connections to each striatal cell with neighboring cells receiving distinct sets of inputs seemed more likely. Given certain quantitative measures of cell density, dendritic spread, and synapse numbers along with the anatomy of individual cortical neuron terminal areas, it was concluded that approximately 5000 cortical cells synapse with an individual striatal cell, with an overlap of inputs to the nearest neighbor of less than 10% (Kincaid et al., 1998; Wickens and Arbuthnott, 2010).

Such a sparse representation meant that chances of finding connected pairs of cells in slices or *in vivo* was small. Although it is possible to occasionally record neuronal pairs in slices, striatal neurons are usually silent in such preparations and the persistent depolarized states called UP states are absent (Rutherford et al., 1988; Kawaguchi et al., 1989; Calabresi et al., 1997a). Since action potentials *in vivo* invariably arise from such depolarized episodes, it seemed important to find a preparation where most importantly the UP and DOWN states were present and where there was furthermore a sporting chance of finding connected pairs of cells. Procedures to enhance “spontaneous” activity include: (i) reduction of magnesium concentration in the perfusate (Calabresi et al., 1992, 1997b); (ii) treatment of slices with NMDA (Vergara et al., 2003); and (iii) a combination of both strategies. Another strategy involves culturing several slices from embryonic brain to in “organotypic” cultures having both cortex and striatum together in connection (Plenz and Kitai, 1998; Tseng et al., 2007). This system is spontaneously active but has many more inhibitory interneurons than normal (Ostergaard et al., 1995). Although much has been learned about corticostriatal activity from these preparations, we wanted a system with not only a more normal complement of interneurons, but also the accessibility and the spontaneous activity observed in organotypic cultures.

Cultures containing both cortical and striatal neurons mixed together appeared to represent such a preparation; however, although spontaneously active, (Arbuthnott et al., 2005) cholinergic interneurons were missing and more importantly, they exhibited connections that were never observed *in vivo* i.e., inhibitory striatocortical and corticostriatal connections. A solution to the lack of cholinergic interneurons in cultures of cryopreserved neurons became possible once we added them back successfully from younger embryonic dissections (Schock et al., 2010). Although it did not appear to be a problem in organotypic cultures (Plenz and Aertsen, 1996), inappropriate connectivity remained a problem for us (Randall et al., 2011). Connections from striatal to cortical cells that never occur *in vivo* confounded our measurement of typical corticostriatal synaptic strength (Randall et al., 2011).

While trying to solve problems of inappropriate connectivity we applied a method that allowed plating of cortical neurons in a compartment and striatal neurons in another. After removal of the wall separating the compartments, cells eventually grew connections between the two regions. Visually, cortical cells grew out processes toward striatal neurons and vGluT1 positive synaptic structures were clearly observed among striatal cells. Further, neurons cultured on multielectrode arrays (MEA) demonstrated cortical spontaneous activity passed onto striatal neurons. Cutting the culture along the line between the two regions yielded little change to cortical activity, but drastically reduced striatal activity.

## Methods

### Neuronal cultures

Cryopreserved (QBM Cell Science Inc., Canada) mouse neurons (E14–15) were plated onto hydrophilic 35 mm microdishes (Ibidi, Germany) or multielectrode arrays (MEA; Multi Channel Systems) within two different compartments (culture insert, Ibidi, Germany). Cortical neurons were plated at concentrations of 600 cells/μl and striatal neurons at concentrations of not less than 1000 cells/μl.

MEA's were steam sterilized (SX300, Tomy, Japan) and rendered hydrophylic by a 30 s treatment in a plasma cleaner (Hitachi High Technologies, SPC-50). Surfaces of microdishes or MEA were pretreated overnight with poly-D- or poly-L-lysine (Sigma) respectively. For the first 18 h in culture the insert was kept in place and medium (NbActv4, Brain Bits LLC, UK) was supplemented with 5% heat-inactivated horse serum (Invitrogen). Cultures were maintained in an incubator at 37°C, 5% CO2/95% O2. Half culture medium was exchanged twice a week with 1% penicillin/streptomycin (Invitrogen) in fresh medium. To reduce evaporation and maintain gas exchange each MEA was individually sealed with a protective cap (Multi Channel Systems) and placed on a Petri dish (85 × 20 mm) with a small glass dish containing 2 ml of ultrapure water (Millipore, TMS-006-B).

### Transfections

In spite of most of our experience being with cryopreserved neurons (Schock et al., 2010) we initially found transfection difficult with standard methods and used instead striatal and cortical primary cultures of neurons isolated from embryonic mice (E17). Tissue was digested in trypsin and plated in ibidi dishes as described above. Neurons isolated with these procedures were transfected with pLenti-hChR2-GFP (Deisseroth, K. Stanford, USA) on day 1 *in vitro* and the insert removed the following day.

### Whole cell patch recordings

Neurons (15–25 DIV) were recorded with borosilicate glass micropipettes heat polished to obtain direct current resistances of 4–6 MΩ. Micropipettes were filled with an internal solution containing in mM: 115 KH_2_PO_4_, 2 MgCl_2_, 10 HEPES, 0.5 EGTA, 0.2 Na_2_ATP, and 0.2 Na_3_GTP. A microelectrode amplifier with bridge and voltage clamp modes of operation (Axoclamp 700B Molecular Probes, USA) was used. Conventional characterization of neurons was performed in voltage and current clamp configurations. Access resistances were continuously monitored and recordings with changes in resistance greater than 20% were aborted. pClamp software was used for data acquisition and analyses were performed using Origin (version 8.6, Microcal, Northampton, MA). To elicit synaptic potentials, channel rhodopsin was excited using an LED light-source providing a maximum of 15 mW power (OptoLED, Cairn Research Co., U.K.). Theta burst stimulation consisted of 6 trains of 5 pulses at 4 Hz delivered at 10 s intervals.

### Multielectrode array (MEA) recordings

MEA with 60 electrodes of 30 μm in diameter, spaced 200 μm from each other were used to record spontaneous network activity starting at 21 days-*in vitro*. N-methyl- D- aspartic acid (NMDA, Sigma-Aldrich) was used to increase corticostriatal bursting (Vergara et al., 2003). Typically cells were recorded in their own incubation medium or in artificial cerebrospinal fluid (ACSF; in mM: 136 sodium chloride; 5 potassium chloride; 1 magnesium chloride; 2.5 calcium chloride; 10 Hepes-sodium; 10 glucose) and no differences in recorded activity were seen between the two media. MEA temperature was kept at 37°C by a temperature controller (Multichannel Systems TC01/02 Rev D). Cell activity from each electrode was fed into the commercial 60- channel amplifier (Multi Channel Systems, MC-rack, Reutlingen, Germany) at 25 kHz sampling rate. To keep medium pH stable cells continually received carbogen (5–95% CO_2_/O_2_) bubbled in sterile water during recordings.

We examined three experimental conditions. First neurons were continuously recorded in their incubation medium for 3–6 min. Second, NMDA (250–300 nM) was then added to the medium and recordings were consequently performed for 3 min every 5 min. Finally a cut between cortical and striatal neurons was performed 15 min after NMDA was first introduced. At least 10 min elapsed before effects of the cut were recorded.

### Immunocytochemistry

**Figure 1:**
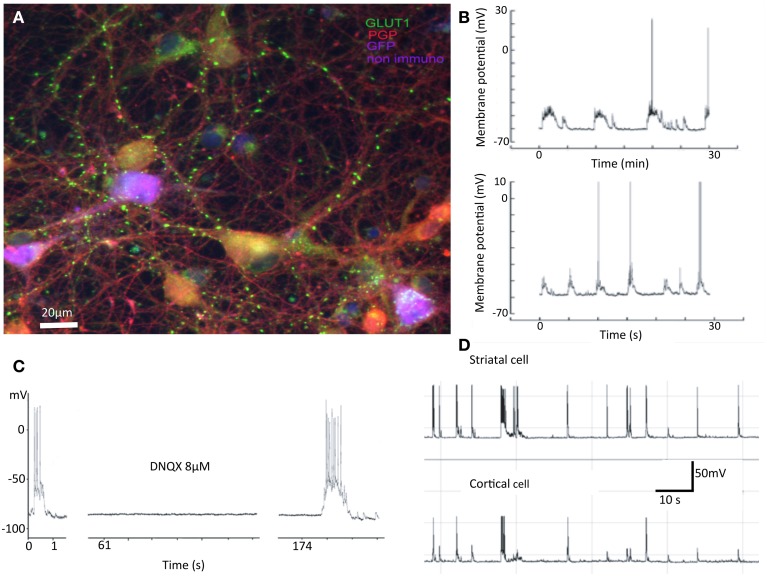
**Summary of previous results**. Properties of mixed cultures that are also seen in separated cultures include: **(A)** Synapses are formed between neurons from cortex and striatum. In cultures cortical and striatal cells do not have the obviously different morphologies expected from *in vivo* investigations. In this false color image, cortical cells are purple, striatal gold, and inhibitory interneurons red. The green dots are glutamatergic boutons stained with antibodies against vGluT1. **(B)** Spontaneous activity in striatal cells shows “UP” states with action potentials riding on them. **(C)** Such “UP” states are abolished by application of AMPA receptor antagonist DNQX. **(D)** Activity of cortical and striatal cells recorded simultaneously are closely related in time (Randall et al., 2011).

Separate cultures were maintained in a 96 well plate for 36 days *in vitro* fixed (in 4% paraformaldehyde with 14% picric acid) and stained with antibodies to VGluT1 (Millipore) and PGP9.5 (Sigma) and appropriately colored secondary antibodies were applied. The cortical cells in these cultures (Figure 1, see Randall et al., 2011 for details) were from a GFP mouse (UBC driven 6 his-ubiquitin/GFP mouse, Tsirigotis et al., 2001) so that cortical cells were already fluorescent.

### Data analyzes

MEA data was initially analyzed with the program Spanner XBD (version 3.5.1, Result GmBH, Tonisvorst, Germany) spikes were extracted when the raw signals overcame a threshold set at 8 times the standard deviation of the root mean square noise. The recorded spike trains were processed at spike and burst levels.

We also transferred MEA output directly to MatLab. Recorded activity was first filtered (400 Hz–8 kHz) to exclude bias and local-field potentials (LFP). Spikes were then detected by counting the number of peaks that exceed the value of ±4σ, where σ is the standard deviation of the signal estimated over non-overlapping windows of 100 ms (2500 samples). Typically in each MEA plate there is a subset of electrodes that dominates total activity.

To summarize results we first computed separate averages of activity of all cortical and striatal cells for each individual MEA (**Figure 5A**) and then performed an analysis of correlations between regions of the MEA as discussed below (**Figure 5B**).

### Corticostriatal correlation function

Cell activity was first studied by calculating the average peak counts of cortical (c) and striatal (s) electrodes:

$\text{c}({\text{t}}_{\text{k}})=\frac{1}{{\text{N}}_{\text{c}}}\sum_{\text{i}\in \text{C}}{\text{v}}_{\text{i}}({\text{t}}_{\text{k}})$, $\text{s}({\text{t}}_{\text{k}})=\frac{1}{{\text{N}}_{\text{s}}}\sum_{\text{i}\in \text{C}}{\text{v}}_{\text{i}}({\text{t}}_{\text{k}})$ where v_i_ (t_k_) is the number of peaks detected by the ith electrode within time-bin t_k_ and N_C,S_ are, respectively, the electrode numbers of numbers of active cortical and striatal electrodes.

The corresponding 2 × 2 correlation matrix is then:

(1) $$\text{ R}=\left(\frac{\bar{\delta \text{c}\delta \text{c}}}{\delta \text{s}\delta \text{c}}\frac{\bar{\delta \text{c}\delta \text{s}}}{\delta \text{s}\delta \text{c}}\right),\bar{\delta \text{c}\delta \text{s}}\equiv \frac{1}{\text{K}}\sum_{\text{K}=1}^{\text{K}}(\text{c}({\text{t}}_{\text{k}})-\bar{\text{c}})(\text{s}({\text{t}}_{\text{k}})-\bar{\text{s}})$$

Where $\bar{c}=\frac{1}{K}\sum_{k=1}^{K}c(t_{k})$, $\bar{s}=\frac{1}{K}\sum_{k=1}^{K}s(t_{k})$ are the mean values, and K is the number of samples (i.e., total number of time-bins). The correlation coefficient, $\rho \;=\;{\text{R}}_{12}/\sqrt{{\text{R}}_{11}{\text{R}}_{22}}$ was calculated in 100 ms bins (*K* = 1800).

### Mutual information (MI)

MI between electrodes was calculated as:

(2) $$I_{ij}=\sum_{a_{i}a_{j}=0}^{V_{i}V_{j}}f_{ij}(a_{i},a_{j})\text{ log}\{f_{ij}(a_{i},a_{j})/[f_{i}(a_{i})f_{j}(a_{j})]\backslash \}$$

Where *f_i_*(*a_i_*), *f_ij_*(*a_i_*, *a_j_*) are the marginal 1-point and 2-point empirical frequency,

(3) $$f_{i}(a_{i})=\frac{1}{K}\sum_{k=1}^{K}\delta (v_{i}^{k}-a_{i}),f_{ij}(a_{i},a_{j})=\frac{1}{K}\sum_{k=1}^{K}\delta (v_{i}^{k}-a_{i})\delta (v_{j}^{k}-a_{j})$$

In equation (3) *v^k^_i_* stands for activity (i.e., number of counts) of electrode i in time-bin *t_k_*(*K* = 1, 2,…, *K*), *K* in the number of samples, and index *a_i_* (similarly *a_j_*) assumes all the distinctive (integer) values of *v^k^_i_*. In equation (2), *v_i_* = max*_k_*(*v^k^_i_*) denotes the maximal count detected by electrode i, while *a_i_* runs over all possible values (*a_i_* = 0, 1,…, *V_i_*) for which *f*(*a_i_*) is non-zero. To avoid undersampling, the marginals in (2) were calculated in 100 ms bins which, as in the previous case of corticostriatal correlation, yields *K* = 1800 samples per electrode. In order to take into account finite-size effects that could in principle lead to spurious MI > 0 even for factorized (statistically independent) cases, *I*^raw^ in (2) was corrected by subtracting the average MI of a null-model (Weigt et al., 2009), I = *I*^raw^ − *I*^0^. The null model is obtained from 1024 random permutations of the samples for each electrode.

## Results

We have observed that segregated cultures develop synaptic connections and display up-states, spontaneous activity and similar pharmacology to corticostriatal slices. As anticipated because of the presence of cortical cells (Segal et al., 2003), spontaneous depolarizations are blocked by DNQX and APV. When neurons were recorded simultaneously, up states are correlated overall and as reported by Randall et al. (2011) and summarized in Figure 1, it is not clear which neuron is driving the other even in connected pairs.

**Figure 2:**
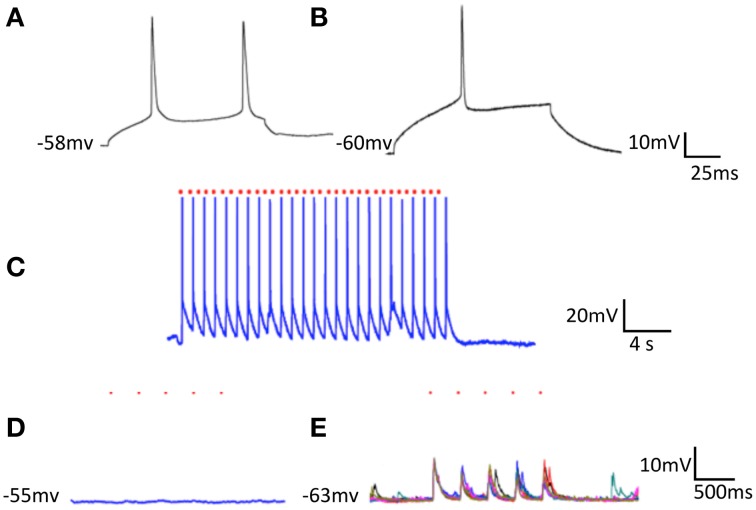
**Optical stimulation of ChR2 expressing striatal cells on cortical neurons plated in separate compartments**. Single cortical **(A)** and striatal **(B)** neurons both depolarized with 80 nA intracellular current. **(C)** Striatal cell (seen in **B**) expressing ChR2 followed every light stimulus (at red dots) for extended periods. **(D)** Optical stimulation of striatal neurons (trains of 5 light pulses repeated 5 times -at the red dots) had no effect on cortical cells. **(E)** Optical stimulation of cortical neurons (same parameters as in **D**) drove EPSPs in striatal cells.

We were able to efficiently transfect cortical neurons with channel rhodopsin2; plate them as before with striatal and cortical neurons in separate compartments; apply light to stimulate cortical neurons; and record responses in striatal cells. Here we report results observed in segregated cortical and striatal neurons in which a group of cells expressed ChR2. In ChR2 expressing neurons the electrophysiology was similar to that seen in slices. Cortical cells had a slightly more depolarized resting potential than neurons in slices and showed little inward rectification. Striatal cells exhibited the typically delayed spike at threshold arising from the A-type potassium current present in these cells *in vivo* and also displayed more depolarized resting membrane potentials (Figures 1A,B). Some striatal cells were driven reliably by light stimulation of cortical culture area (Figure 2C) and most followed light stimulation of cortical cells with clear EPSPs (Figure 2E). Conversely, when striatal cells expressed ChR2 although they were easily and reliably excited by optic stimulation, cortical neurons were unresponsive (Figure 2). In these conditions inhibitory IPSCs were observed only in striatal neurons (data not shown); no responses were observed in cortical cells.

**Figure 3:**
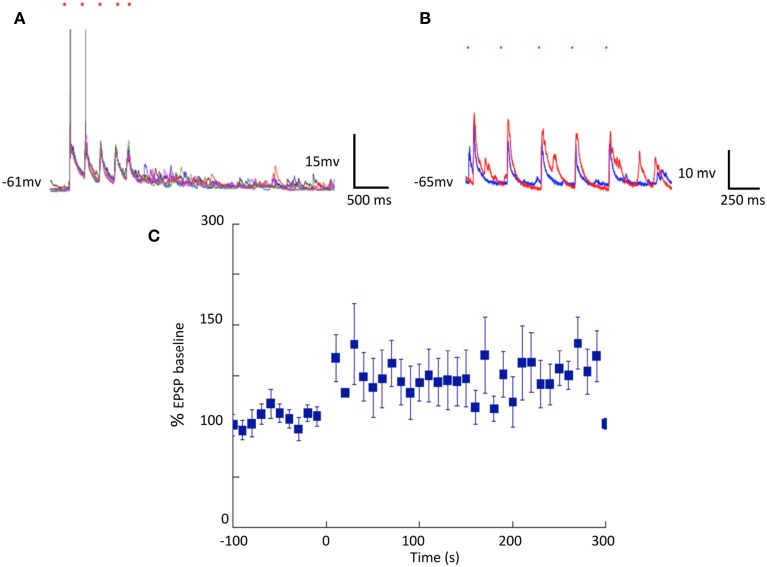
**Cortically induced long-term changes in striatal synaptic responses observed in separated cortical and striatal cultures. (A,B)** Optical stimulation of cortical neurons expressing ChR2: Single pulses (blue traces) and theta-like rhythm (6 trains of 5 pulses at 4 Hz at 10 s intervals red traces) stimulation. **(C)** Summary of EPSP amplitudes before and after theta burst stimulation (*N* = 14).

Longer-term changes in efficacy of light induced stimulation of cortical cells on striatal neurons were also evident. Striatal responses to cortical stimulation were increased in all 14 cells we held for long enough to test. Figure 3 shows individual responses and a summary of all 14 cells averaged including those cells where the change was less obvious than in the example in Figure 3B.

### MEA recordings

Neurons were recorded in three conditions: control (MEDIUM), in the presence of glutamate agonist (NMDA) and following transection of connections (CUT).

**Figure 4:**
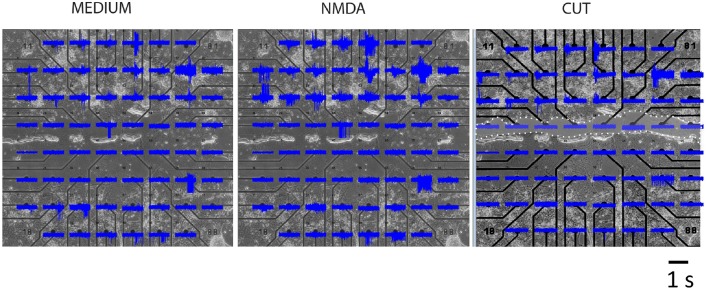
**MEA recordings of a representative segregated culture**. Top four rows contain cortical and lower three rows striatal neurons in three experimental conditions. Area of cut is indicated by white dots and clear shading.

Analyses of MEA recordings indicated presence of a dominant effect of cortical bursts on striatal activity although there was not a one to one correspondence. It seems possible therefore that different clusters of cortical cells gain control of different striatal cell groups from time to time. Not all electrodes were active in striatal compartment and most active electrodes were lost following a knife cut between the two regions (Figure 4).

Many physiological characteristics of the *in vivo* corticostriatal system are present in our cultures and can be deduced from the MEA. We computed two different kinds of mathematical summaries.

**Figure 5:**
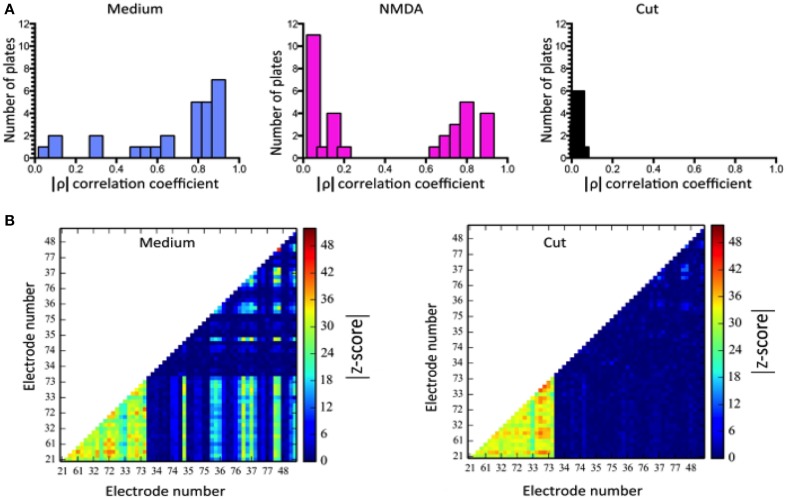
**Correlations of regionally averaged potentials for all plates and the mutual information between electrodes for a representative experiment. (A)** Average correlation between electrode activity averaged over the cortical region and electrode activity averaged over the striatal region (see Figure 4). **(B)** Only half of the electrode-electrode mutual information values are displayed since this quantity is symmetric. In medium there are many different z-scores across electrodes. In medium there are many different z-scores across electrodes. Left hand panel (Medium): lower left quadrant displays mutual information between cortical electrodes; upper right quadrant striatal electrode mutual information; lower right quadrant mutual information shared between the compartments. Right-hand panel (Cut) displays the dramatic fall in information within the striatum and its complete absence between regions. The slight increase in the cortical region is likely due to the intervening NMDA application (see panel **A**) rather than a direct consequence of the cut, but its source remains to be examined in further work. Details of similar analyses in other plates in this study are illustrated in Supplementary Figure 1.

We computed correlations between all the electrodes. The correlation oefficient between cortical and striatal activity, $\rho =\bar{\delta c\delta s}/\sqrt{\bar{\delta s^{2}\delta c^{2}}}$ calculated in 100 ms bins is shown in Figure 5. The differences between MEDIUM, NMDA and CUT are clear. A threshold value, was obtained by random data shuffling to ensure a p value of 0.005 for noise-level (Mao et al., 2001). When only those events whose activity exceeds θ are counted, it turns out that δ*c*δ*s* = 0 after CUT.

Although these calculations of corticostriatal correlations allowed a way to summarize activity among plates (Figure 5A) we were more interested in measures that might give knowledge about information handling in the networks. Therefore we then estimated mutual information (MI) between electrodes by performing calculations in a 100 ms window moving across pairs of recordings (Equation 2). Data was compared with shuffled versions of the data obtained from 1024 random permutations of the samples for each electrode. This allowed calculation of z-score indicative of how much of the observed MI exceeded random levels. Plots in Figure 5B represent z-scores of every electrode pair in one plate. The bottom left shows information shared between cortical electrodes. The top right shows information shared between striatal electrodes and the bottom corner shows information shared between a given cortical and a given striatal electrode. After the cut information between cortical and striatal electrodes vanishes uniformly and almost all information shared between striatal electrodes vanishes as well.

Data analysis using MI for a representative experiment is shown in Figure 5, and as color plots for all the plates in Supplementary Figure 1.

## Discussion

By plating neurons in separate compartments we have developed a culture that makes it possible to study *in vitro* properties relevant to the corticostriatal network as it exists in brain. Optogenetic techniques allow the driving of one group of cells with light stimulation of transfected ChR2. Cortical light stimulation activated striatal neurons, but striatal stimulation did not activate cortical neurons. To make sure that striatal cells were stimulated by light, recordings in that compartment were performed and revealed clear synaptic inhibitions driven from neighboring neurons. The quantitative analyses of MEA recordings is still under refinement however, the described computations of mutual information and correlation between recorded electrodes are fully consistent with one another. Quantitative evidence of connectedness between striatal and cortical electrodes is observed.

Moreover once the links between the two regions are severed the connectivity is not present. In recent results from the “First Neuroconnectomics Challenge” http://connectomics.chalearn.org/home the winners (Sutera et al., 2014) primarily relied on correlation to unravel the network that had generated the data. However, in contrast to ours, the data they used was synthetic and derived from 1000 identical cells.

Our methods of growing separated neurons in culture have already allowed some interesting pharmacological results. For example, responses to NMDA administration show higher firing rates but only minor effects on the measures of MI. Obviously other features of the system will be worth exploring in these cultures but also in similar cultures developed from other connected regions of brain. The importance of extrasynaptic NMDA receptors (Garcia-Munoz et al., 2015) is one aspect that could shed light on the mechanisms of the “UP” and “DOWN” states characteristic of these cells in anaesthetized animals.

There are other lessons we have learned in passing. The idea that synaptic connections are dictated by the genome of the neurons is true only in special circumstances. Clearly, given the opportunity, striatal cells do make synaptic contact with cortical cells that are excluded in real brains. Developmental studies have indicated that although cortical GABA interneurons are derived from the same site as the striatal cells, they clearly travel along guidance routes that are not available for striatal output neurons. Those neurons do not innervate forward areas of brain; they make connections with each other, in the midbrain and hindbrain but not cortex. Just keeping cortex 500 μm away is enough to reduce this activity to as near zero as we can measure. Cortex, on the other hand aggressively innervates, and indeed is vital for the survival of, the striatal cells. Cortical cells connect not only to striatal cells but also to other cortical cells in the region. The measures we made of pairwise correlations and mutual information are always largest within the cortical area. Indeed the properties of such cortical networks have been extensively studied ever since the early studies of isolated cortex *in situ* in cats (Burns, 1950, 1951, 1955). The firing properties of these isolated systems are interesting in that they sometimes involve many of the cells in the network (Beggs and Plenz, 2003; Wagenaar et al., 2005, 2006; Pasquale et al., 2008; Bologna et al., 2010).

Although the search for convincing brain systems in a dish remains in its early days our cultures have validated some methods for the analysis of connectivity in such cultures. Our new system with a gap between the two compartments makes the connectivity more realistic although its size makes it difficult to find connected pairs of nearby neurons with patch electrodes. This system has the advantage of providing a neuronal monolayer that facilitates transfections, anatomical identification and recordings of individual neurons. Compared to slices this technique requires frequent care and supervision of factors like evaporation and contamination but allows repetitive imaging and recordings of the same culture under different conditions. We are working on methods to identify individual pairs of synaptically connected cortical and striatal neurons to examine the properties of individual contacts. Nevertheless we do have MEA recordings whose analyses will generate testable predictions about the real brain, as well as a plausible explanation for the generation of “up” states in cultures and slices (Garcia-Munoz et al., 2015) that may be important in other areas of brain (Oikonomou et al., 2014).

Thus we have achieved a corticostriatal culture system with readily accessible cells for pharmacological, physiological or genetic manipulations. Effects of specific cell types can be studied by adding other compartments. Future prospects include addition of compartments containing dopaminergic, serotonergic and thalamic neurons that, like cortical neurons, provide input to striatal cells *in vivo*. However, since thalamic neurons, like striatal ones are dependent on cortical input for survival, there remain technical challenges to overcome to achieve such a preparation.

### Conflict of interest statement

The authors declare that the research was conducted in the absence of any commercial or financial relationships that could be construed as a potential conflict of interest.
