# Supplementary material for: Rebuilding a realistic corticostriatal “social network” from dissociated cells
Source: Front Syst Neurosci. 2015 Apr 20;9:63. doi: 10.3389/fnsys.2015.00063 (PMC4403293; doi:10.3389/fnsys.2015.00063)
Supplement: Supplementary file 1 [file Image1.PDF]

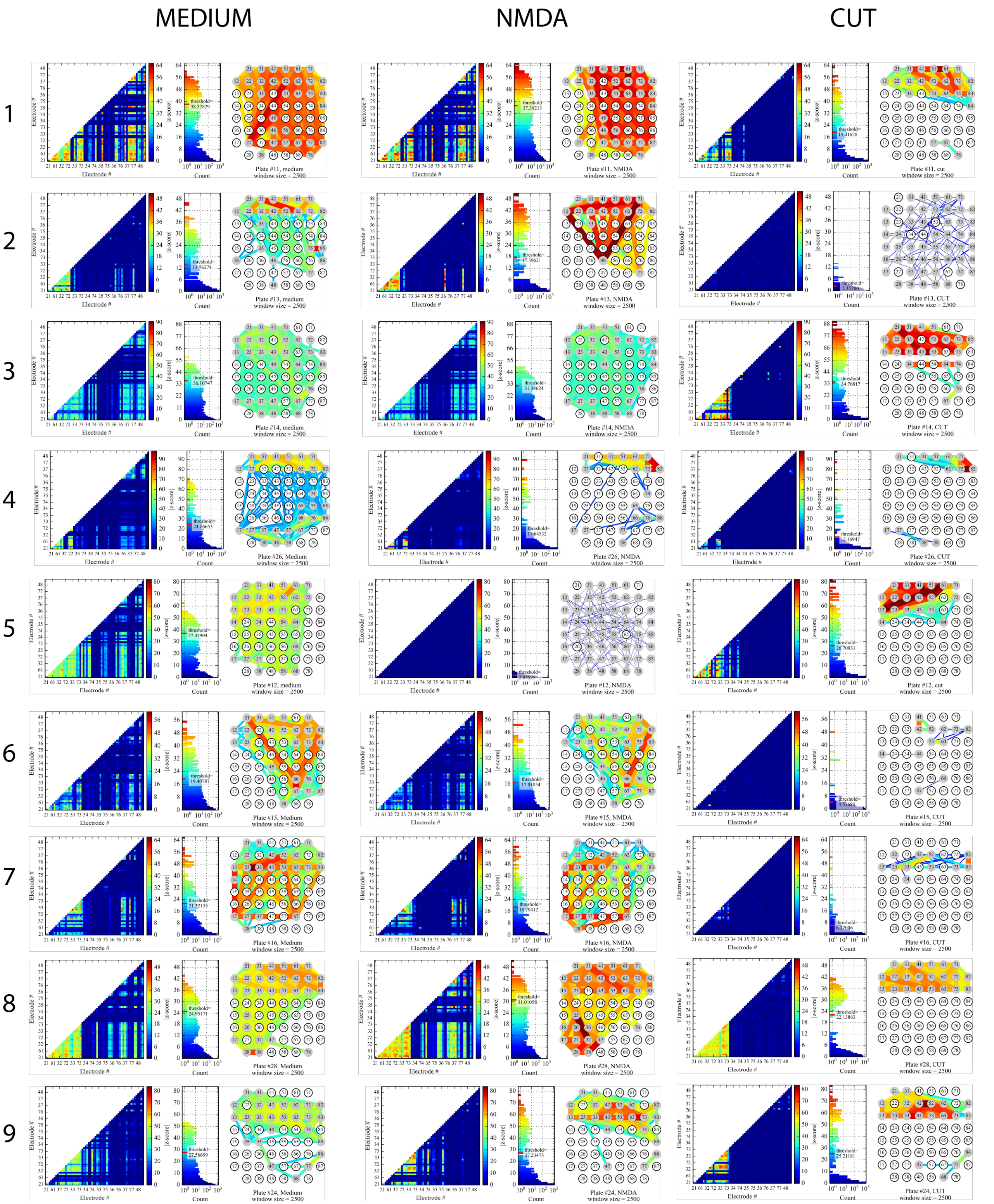

Supplementary Figure 1. Mutual information (MI) analysis of MEA activity of the nine individual experiments. Left column analysis of control activity (medium), middle column analysis of recordings in the presence of NMDA and right column following a separation between the two groups of neurons. Cortical and striatal neurons were over the top and bottom 3-4 rows of electrodes respectively. In general, MI observed in control and NMDA conditions is lost following the cut. Faced with large amounts of data MI has proven a useful tool. Other integrative tools to group, summarize and evaluate all experiments into a single analysis are still needed.
